# Novel Human-Centered Robotics: Towards an Automated Process for Neurorehabilitation

**DOI:** 10.1155/2021/6690715

**Published:** 2021-01-29

**Authors:** Meenakshi Devi Parre, B. Sujatha

**Affiliations:** ^1^Department of Mechanical Engineering, IIIT, RGUKT, RK Valley, Kadapa, Andhra Pradesh, India; ^2^Department of Zoology, GDC, Rayalaseema University, Nandyal, Kurnool, Andhra Pradesh, India

## Abstract

The global requirement of patient rehabilitation has surged with time due to the growing number of accidents, injuries, age-related issues, and other aspects. Parallelly, the cost of treatment and patient care also increased in a manifold. Moreover, constant monitoring and support for the patients having physical disabilities have become an ongoing challenge to the medical system. Robotics-based neurorehabilitation has reduced the human error while assisting such patients, precisely interpreting the signals, and communicating to the patient. Gradual precise application and improvement of the technology with time yielded a novel direction for patient care and support. The interdisciplinary contribution of many advanced technical branches allowed us to develop robotics-based assistance with high precision for the upper limb and the lower limb impairments. The present review summarizes the generation and background of robotic implementation for patient support, progress, present status, and future requirements.

## 1. Introduction

The human being is considered as one of the most complex machines on the planet Earth. However, the synchronicity between multiple systems, their organized interactions, and the appropriate responses to the environmental stimulations are astonishing. Managing multiple complex responses simultaneously appreciates the level of multidimensional organization and excellent coordination among the systems. All these coordinations are managed and maintained by the web of the neural system that generates and transmits numerous signals and responses in split seconds; thus, it helps the system to function in a smooth and effective manner.

However, there are certain times when an unwanted interruption occurs due to any musculoskeletal or neurological disease, injuries, amputations, and similar reasons. Such painful conditions hamper the daily and regular movements and functionality of the patients and hence stall the normal life. Depending on the severity of the conditions, the time, process, and steps of rehabilitation differs. Therefore, neurorehabilitation is attributed to the highly complex process or systematic medical effort to help in recovering, restoring, or minimizing the losses from a nervous injury with all possible normal functionality in a human being [1]. Certain health issues remain a challenge for the patient, doctor, caregivers, and family members. Therefore, the rehabilitation process often turns out to be prolonged and extensive.

**Figure 1:**
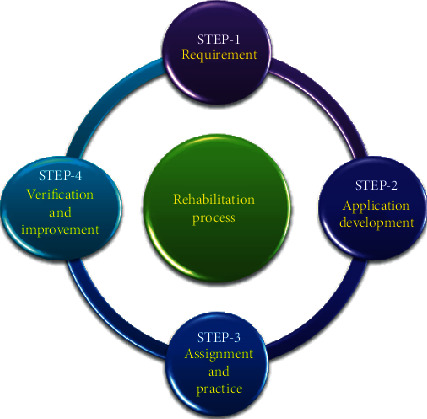
Cycle of the rehabilitation process and associated precise protocol development and implementation.

The need for neurorehabilitation was extremely felt important after World War II when several injured soldiers who averted death and infection required rehabilitation for their spinal cord and head injuries. Since then, the process of neurorehabilitation has observed a gradual development and novel applications for better therapies and applicability. Many major conditions can cause various degrees of physical disability such as multiple sclerosis, Alzheimer's disease, Parkinson's disease, neuropathies, apart from the severe spinal cord, head injuries, and stroke. Guillain–Barre syndrome is another major cause that leads to restricted physical movement and often requires extensive rehabilitation and related patient care. Often, a complex neurorehabilitation process requires a team of professionals or experts to work together for optimum patient outcomes. Such teams may include specialized doctors and nurses trained in a particular type of rehabilitation therapy, speech therapist, patient and family counselors, and other experts depending on the case and necessity. The cycle of the rehabilitation process is implemented as mentioned in Figure 1.

In the first step, evaluation for the specific requirement is inspected, followed by the requirement of specific application development. Later, the developed application is assigned into trial practices, if found suitable and effective; after verification, the developed application or protocol is continued, else further improvement is done depending on the necessity.

## 2. Major Types of Health Conditions Require Rehabilitations

### 2.1. Stroke

The primary cases are majorly due to stroke, head injury, and spinal cord injuries which are allotted neurorehabilitation therapy on a short-term or long-term program based on the degree of treatment required. Often, stroke survivor patients require rehabilitation therapies due to partial or complete paralysis and decline or loss of functionality of a specific side or limb of the patient [2]. Functional limitations, mostly hemiparesis or hemiplegia, require such rehabilitation therapies. The patient's condition determines the possibility of therapies; for instance, patients with right cerebral infarct and left hemiplegia will be inspired to attempt to work as a right-hander rather than left-hander; the emphasis from the therapist is performed towards making the patient as most independent as possible. In such stroke patients, the majorly affected body parts are the upper limbs such as the arms and hands compared to the lower limbs such as the legs. Therefore, with the help of a suitable walker, the patient may be able to walk. Thus, a specific type of disease or condition can affect specific limbs or organs which can be treated accordingly. Patients' outcomes are constantly assessed and monitored using various scaling measures to understand the gradual improvement and estimate the time required for maximum improvement to attain. Degree of impairment determines the duration of recovery; for instance, only motor involvement can be improved comparatively quickly rather than involvement of sensory nerves and hemianopsia. Several ways are implemented depending on the stroke survivor patient's condition. Modern therapies may include external noninvasive brain stimulations, improvement of the brain plasticity as part of the therapy [3], application of sophisticated and well-coordinated brain-machine interface [4], and real-time application of robotic devices.

### 2.2. Head Injury

This type of injury requires the extensive rehabilitation program as serious head injury may cause physical, cognitive, learning, and movement impairments and can sustain life long, and it may require crucial support from the family and community members. Thus, such impacts cause personality alteration of the patient and require proper and detailed counseling of the patient and the family members. Head injury conditions are associated with several other factors during the rehabilitation process such as emotional and biopsychosocial aspects. A variety of patient outcomes have been standardized and reported to understand the recovery of head injury. Sleep disturbances and countering such issues remained an important aspect of accelerated recovery for closed head injury patients. Modern instruments and devices such as actigraphy and polysomnography are extensively used to monitor the neurorehabilitation process and progress the patient outcomes [5, 6].

### 2.3. Spinal Cord Injury

Spinal cord injury can lead to a severe degree of disability that may include paraplegia, impaired voluntary and involuntary reflexes, impaired bowel movement, and others. Such kind of situation may arise from an accident or similar incident. The complexity of the problem depends on the degree and area of injury in the spinal cord such as, for instance, tetraplegic patients having severe cervical injury may have much complicated problems. The impairment in the motor function varies depending on the spinal cord region affected, such as patients having an injury in the C5–C7 may have functionality in the upper arm. For spinal cord injury associated rehabilitation processes, several modern efforts are becoming fruitful such as application of brain-machine interface to stimulate the spinal cord specifically for precise outcomes and functionality [7]. Other simple applications, such as passive cycling [8], the aid of virtual reality to invoke the reflexes, are being implemented to have better outcomes and patient benefits [9]. Precise biomarkers such as cutaneomuscular spinal reflex activity are also considered to understand the degree of motor dysfunction and determine the appropriate neurorehabilitation measures [10].

### 2.4. Disease Conditions

Several disease conditions such as multiple sclerosis, Parkinson's disease, peripheral neuropathies, and Alzheimer's disease often sought neurorehabilitation. Numerous studies have been conducted in these aspects. Messinis and colleagues recently conducted a randomized controlled trial on the impact of the computer-based cognitive neurorehabilitation for the secondary progressive multiple sclerosis (SPMS) patients [11]. They have reported that training by RehaCom™ software was effective for the rehabilitation of the SPMS through reduction of the cognitive fatigue, improvement of cognition, and quality of life in the considered patients [11]. Similarly, neuroplasticity is also considered as a part of the neurorehabilitation program for multiple sclerosis patients [12]. Recent advances in the treatment of cognitive deficit multiple sclerosis are reported by Sokolov et al. [13]. Computerized neuropsychological training and cognitive-behavioral therapies use disease-combating drug combinations to attenuate the disease progression or improving cognition being used simultaneously. Similarly, several studies with relation to neurorehabilitation have been conducted to treat neuropathies [14], Alzheimer's disease [15], and Parkinson's disease [16, 17].

### 2.5. Requirement of Interdisciplinary Approach and Automation

All these present research studies on the neurorehabilitation of various complex health conditions suggest that there is an urgent requirement for an interdisciplinary approach [18] and automation of the rehabilitation process [19]. Certain factors are encouraging us to adopt the automation for the neurorehabilitation process. Precise identification of the physical, emotional, behavioral signals of the patients, the requirement of constant and round the clock monitoring of the patients, accurate diagnosis and deploying the appropriate therapeutic steps where the patient is unable to communicate the conditions, and long-term tireless physical and systematic support to the patient are such important factors that are leading towards the adopting automation in neurorehabilitation.

### 2.6. Automation: A Step towards Using Robotics for Neurorehabilitation

#### 2.6.1. Automation in Patient Evaluation Protocol

The success of computer-based condition evaluation and systematic training and therapies about neurorehabilitation is another vital reason for embracing automation in the neurorehabilitation process. In the 1960s, such efforts were already under consideration, and there was some initial success as well. Brain injuries were assessed successfully through the computerized Wisconsin Card Sorting Test that helped in avoiding human mistakes. Functional memory was evaluated for the subjects using the Rivermead Behavioral Memory Test (RBMT) that examined the daily functions of a subject in relation to their functional memory. The online version of the same test (OL-RBMT) was effectively implemented and automated to evaluate a large number of patients accurately.

#### 2.6.2. Automation in Physiological Signal Interception, Interpretation, and Behavior

The progress of the initial automation success for patients' evaluations allowed the researcher to upgrade the automation and to establish the human-machine interactions and understand the physiological aspect of the neurorehabilitation process through a machine-based automated way devoiding the possible manual error for better treatment and recovery. Some specific important progress is mentioned in the following section.

Quantitative electroencephalography (QEEG) is a complex mechanical procedure that includes a great number of tools and techniques. This system captures the high-speed sequences of cognitive processes that happen in a fraction of a second in a specific order. Thus, automation of such a process can aid in tracking the patient's response towards a particular normal of applied stimulation [20]. Such a sophisticated QEEG system has been automated and successfully implemented for delayed cerebral ischemia (DCI) analyzing the alpha delta ratio and relative alpha variability [21].

Evaluation of brain functions and cognitive abilities are being tracked through the eye tracking system, a noninvasive and effective procedure to monitor the patient's condition [22, 23]. To improve the prediction accuracy and precision of the eye tracking system, many important variables are considered such as gaze points, fixation, and relevant parameters. Such inputs allow analyzing the processing of stimulus sequence even in a multistimulus ambiance. Therefore, this kind of complex signal transduction and interpretation allowed us to evaluate the patient's responses against environmental stimulations and their improvement with time.

However, furthermore, the improvement was required to increase the interaction between the patient and the doctor, caregiver, or therapist. Virtual reality (VR) played a pivotal role in this context and aided to improve the neurorehabilitation program altogether. The VR system has been effective for stepwise cognitive training of the patients, especially those having difficulties performing daily sequential activities due to stroke or similar health issues [24]. Similar other memory training and practices are also reported using the ability of VR.

Besides, specialized cognitive training and special sequence-based regular activity training modern sensor-based methodologies are being implemented to support the patients who require mild, moderate, or serious support for their survival and activities. Automation has played an essential role for the patients and the caregivers at various stages of neurorehabilitation and helped the patients to compensate for their deficiencies upto a certain extent. However, modern robotics and artificial intelligence-based methods can yield further. Novel developments such as the decision support system (DSS), based on AI [25], automatic assessment system (AAS), deep learning system (DLS), and robotic rehabilitation systems (RRS) can become a game changer in this aspect. The helpful feature of artificial intelligence is the learning and training system for accurate response. Such facilities enabled to render help from AI-derived socially assistive rehabilitation robots that can understand the verbal instruction and respond with precise feedback for the patient [26]. A combination of advanced algorithms such as an artificial neural network (ANN), fuzzy logic, and inverse kinematics was used for the development of a novel video game-based neurorehabilitation system for multiple sclerosis where the inputs from the patients and the outputs were predicted using these algorithms with higher accuracy [27]. Application of ambient intelligence for monitoring and evaluating neurological responses has been reported earlier [28].

## 3. Application of Robotics

### 3.1. Prior Assessment

Implementation of robotics depends on the prior assumptions and detailed evaluation of a specific patient condition. It requires application establishment with precise planning based on the in-depth analysis of the patient condition data. Individual problems are analyzed based on the specific nature of the problem, influencing factors involved, prognosis, type and severity of the impairment, mechanical and systematic limitations, and social and environmental issues. To scale and detect the level of impairment, activity, and participation, an array of tests and clinical assessments are completed.

Movement, balance, and cognition are evaluated using many scales including but not limited to the Beck Depression Inventory, Mini-Mental State Examination, Behavioral Inattention Test, clock drawing test, Fugl-Meyer assessment of motor recovery after stroke, Orpington Prognostic Scale, and Montreal Cognitive Assessment. All these evaluations are performed to understand the degree of impairment in the patient. Similar to impairment assessments, another array of scales are used to understand the activity range of the patient; for instance, the Barthel Index, Action Research Arm Test, Timed “Up and Go” Test, Chedoke-McMaster Stroke Assessment, and Rivermead Mobility Index are extensively used depending on the necessity. Depending on the patient's emotional involvement and willingness, participation is evaluated using another set of standard scales. All these exercises are directed towards reducing the patient's uncomfortable situation and render a comparatively active independent daily life. Thus, the goal remains disability reduction, improved activity, maximum social acceptance, and participation. Robotics are successfully implemented to enhance such goals and relieve the impairment of the patient or improve the activity as much as possible.

### 3.2. Robotics in Upper Limb Rehabilitation

Upper limbs are mostly affected by the stroke survivors. Hence, a careful and extensive rehabilitation approach is required for their recovery and daily activity. Experimentation with the elbow-shoulder manipulator and assessing the stroke-affected patients suggested that robotic device support improved the patients' motor functions concerning the arm movement considerably when compared to the patients who not availed such devices. A meta-analysis on the application of distal and proximal arm robotics suggested that robotic-assisted efforts improve the motor function recovery of the upper limb function significantly for poststroke patients. Another similar investigation also suggested that robot-assisted therapy for the paretic upper limb (RT-UL) in poststroke patients significantly increases their practice repetition and improves the patient recovery substantially [29]. Another randomized controlled trial study also reported that robot-assisted therapy (RT) for the wrist and forearm movement can improve the motor functions and enhance the functional performances in chronic stroke patients [30]. A study reported that chronic stroke patients displayed fast clinical outcomes using robot-based assessment via the kinetic and the kinematic macrometrics that were developed for the upper limb functionality [31]. Apart from the improvement of the impaired upper limb functions in the patients, competitive machine learning-based methods were also able to predict the upper extremity Fugl-Meyer assessment (UE-FMA) at the posttherapeutic intervention stage for the chronic stroke patients [32]. Furthermore, another pilot study conducted on upper limb motor activity recovery suggested that robotic interventions and support stimulated the improvement of the impaired function better than the conventional therapy [33]. Specific examples of the hand and upper limb rehabilitation using robotics are discussed in the following section.

### 3.3. Hand Rehabilitation

Soft robotics systems are simpler in design, successful in enhancing safety, efficacious, and portable. The major applications are in hand rehabilitations [34]. Assessment based on the control unit and wearable orthosis suggested that several unique designs of soft robotic systems have been developed in recent years.

#### 3.3.1. Upper Limb Exercises Using Robotic Devices

Care of an injured patient often requires long-term therapeutic sessions and hospital stay. The expenditure also surges depending on the therapeutic needs and duration of the hospital stay. Home-based robotic devices may become beneficial for automated robotic therapeutic approaches and ease of the process with required accuracy and precision. A recent report suggests that a patient in a condition to take the advantage of home-based Computer-Assisted Arm Rehabilitation (hCAAR) may improve their upper limb movement with minimal supervision. Availability and functionality of such devices can boost the psychological confidence and the independence of the patients and may reduce the efforts of the caregivers at home [35].

#### 3.3.2. Upper Limb Exercise Outcome Measures

Long-term therapeutic sessions require occasional outcome measurement to understand the gradual improvement of a patient undergoing rehabilitation therapy. However, the evaluation of the outcomes becomes cumbersome as the direct feedback from the patient less. Such a problem further intensifies while applying a robot-assisted exercise trial (RAET). Therefore, a defined, standardized, and acceptable outcome measurement approach should be applied for a better understanding of the therapeutic benefits for the patients. A recent study by Sivan and colleagues suggested that applying different outcome measures recommended by the International Classification of Functioning, Disability and Health (ICF) was found beneficial and appropriate [36]. Several ICF scales used to assess the chronic severity and weakness improvement are effective, and certain scales such as Fugl-Meyer (FM), Modified Ashworth Scale, Motor Status Score, and Kinematic measures could be useful to evaluate the outcome measures effectively along with other measurements such as the Action Research Arm Test, ABILHAND, Functional Independence Measure (FIMTM), and Wolf Motor Function Test.

#### 3.3.3. Upper Limb Exoskeleton Control

Cerebrovascular injuries affect the motor neuron function in patients. Such patients require extensive rehabilitation therapies that may include functional electrical stimulation (FES) devices, artificial exoskeletons, and supports with brain-machine interfaces (BMC). Depending on the injuries and the damages, a combination of these systems could be applied for the best plausible rehabilitation of the patients. A combined approach with hybrid upper limb exoskeleton and brain-machine interface displayed promising results with increased accuracy in a recent study [37]. Further experimentation may allow understanding of the increased benefits in this context.

#### 3.3.4. Upper Limb Exoskeleton Control Strategies

Apart from the mechatronics of the exoskeleton devices used for neurorehabilitation, understanding and categorization of the control systems are essential to understand the qualitative benefits and in the improvement of the quality of life in the patients. A better control interface may have excellent sensors with the best possible sensitivity, may support a higher degree of freedom (DOF) for limb movement, should consider more number of contact points for better movements, and should have superiority in assistance, correction, and resistance [38].

#### 3.3.5. Robotics in Lower Limb Rehabilitation

In continuation of the several examples of robotics-assisted improvement of upper limb functionality for better neurorehabilitation in patients, applications are also reported abundantly for the lower limb functionality and activity improvement through robotics. Such a robotic system follows different rehabilitation principles such as foot-plate-based gait trainers, overground gait trainers, treadmill gait trainers, and ankle rehabilitation systems (stationary or active foot orthoses) [39]. In most of the cases, these kinds of robotic-assisted training conducted for the spinal cord injury affected lower limb function recovery. Several others such state-of-the-art robotic systems for locomotor training are available for the patients having spinal cord injuries such as Ambulation-assisting Robotic Tool for Human Rehabilitation (ARTHuR), Pneumatically Operated Gait Orthosis (POGO), and Pelvic Assist Manipulator (PAM) [40]. ARTHuR aids in evaluating stepping in a treadmill and generating required feedback. On the other hand, POGO and PAM are used for leg-robot designing and pelvic motion control, respectively. Equivalent to these sophisticated robotics-based movements, balance, and posture and motor coordination rectification systems, several others such as the Active Leg Exoskeleton (ALEX) and LOPES (LOwer-extremity Powered ExoSkeleton) are available. The former is a powerful leg orthosis that focuses on the correcting and accurate motion of the hip and knee joints, and the later supports walking movement in a treadmill. Several foot-plate-based gait trainers such as Gangtrainer GT I, HapticWalker, and others are available for effective training and impairment recovery of the patients. Several overground gait trainers including KineAssist, WalkTrainer, and ReWalk are available to support the patient to overcome their spinal cord injury-related impairment. Several advanced robotic support also helps in performing a variety of activities such as hybrid assistive limb (HAL). Several other gaits such as stationary gaits, foot orthoses, and the rehabilitation system for the ankle and knee are available depending on the functional necessity and financial budget. However, even though most of these robotic systems are precise and responsive to the user requirement, yet there are grounds for technical and supportive improvement for personalized utility. Moreover, all these robotic gaits are being used as a short-term or long-term supportive measure for the patients, and no clinical standardization has been decided globally yet.

### 3.4. Spinal Cord Injury (SCI)

Serious spinal cord injury affects and reduces the functional capacity of a patient through compromised neuromotor functioning; hence, the patient may have problems in gastrointestinal, urinary, cardiorespiratory, osteomioarticular, and other functional restrictions. Advanced applications of robotic-assisted gait are successful in improving the reflexes, feeling and sensitivity, electromotor and neuromuscular activity, independence and flexibility in limb movements, and psychological confidence. Such applications of robotic gaits also helped in reducing spasticity and pain in many patients [41].

## 4. Modern Advanced Applications

Understanding the neurophysiological mechanism in detail has been possible due to the advancement of science and technology. Such knowledge will lead to a better understanding of the normal neuromuscular system [42, 43]. Intricate knowledge of the physiological, biochemical, and genetic aspects of neuromuscular functioning will aid in understanding the minute mechanism of the system. Hence, it may help in customizing the requirement of the patient with the help of modern science and technology.

The advancement of robotics further inspired the global effort to develop an integrated system with the utmost precision and flexibility for patients with varying degrees of impairments. The growing number of cases of chronic disabilities and ever-growing cost of patient care allowed the global researchers to develop large scale multidimensional and multinational projects such as the “Cognitive Assistive Living Ambient System—COALAS” where 1.6 Euro have been funded [44]. The project goal is to implement modern robotics approaches integrated with information and communications technology (ICT) and render a better flexible solution to the patients in need and improve their quality of life. For the patients who are in a wheelchair, the project supports the navigation improvement of the electrically powered wheelchairs extensively along with better communication technologies with the environment and others. Another such amalgamation of technologies is the HYPER project [45]. This project implements the hybrid implementation of exoskeletal robots (ERs), motor neuroprosthesis (MNPs), virtual reality (VR), and brain neuro-machine interface (BNMI). Specifically for children who need such robot-assisted technologies for neurorehabilitation, the THERAPIST project was conceptualized that can help in automating the social interactions through robot-assisted motor functioning in children [46]. In this project, the RoboCog system was established that can interact with the patients equivalent to humans, thus developing a robotic-based smooth communication between the patient and the environment. Another vital aspect is proper timely drug delivery through robotic approaches in complex situations. Efforts have been made to build up neurorehabilitation haptic devices for drug delivery [47].

## 5. Future Perspective

The robotics-based applications are showing their extensive promises in the process of neurorehabilitation for the patients and helping them regain their confidence through calibrated training, practice, and increasing their emotional and mental confidence. The state-of-the-art technologies combining biomechatronics, information technology and communication, mechanical engineering, biomedical engineering, and sensor-based approaches have raised the hope in the patients and the caregivers. However, there are several hurdles to overcome in the near future. Improvement of precision and accuracy, flexibility in the mechanical movement of the prosthetics, and development of personalized solutions are some of the crucial technical challenges. Most importantly, the cost-effective manufacture of such products and robotics-based assistive instruments can cater to a wide range of patient populations who are in desperate need of such support.

## 6. Conclusion

Robotics-based neurorehabilitation has proved its technical mettle and applicability in many serious aspects of patient care, surgery, and neurorehabilitation. However, the time has come where a global standard should be determined and a further complete system-based approach has to be taken through large international collaborations to cater to the requirement of a huge patient who is in need. Today, great many options are available for different types of injuries of the upper limbs and lower limbs that may arise due to serious health issues. However, overcoming several technical challenges and financial issues may aid the patient population abundantly.

## Data Availability

The data used to support the findings of this study are available from the corresponding author upon request.
